# Therapeutic Drug Monitoring of Meropenem and Piperacillin in Critical Illness—Experience and Recommendations from One Year in Routine Clinical Practice

**DOI:** 10.3390/antibiotics9030131

**Published:** 2020-03-21

**Authors:** Christina Scharf, Michael Paal, Ines Schroeder, Michael Vogeser, Rika Draenert, Michael Irlbeck, Michael Zoller, Uwe Liebchen

**Affiliations:** 1 Department of Anesthesiology, University Hospital, LMU Munich, 81377 Munich, Germany; ines.schroeder@med.uni-muenchen.de (I.S.); michael.irlbeck@med.uni-muenchen.de (M.I.); michael.zoller@med.uni-muenchen.de (M.Z.); uwe.liebchen@med.uni-muenchen.de (U.L.); 2 Institute of Laboratory Medicine, University Hospital, LMU Munich, 81377 Munich, Germany; michael.paal@med.uni-muenchen.de (M.P.); Michael.Vogeser@med.uni-muenchen.de (M.V.); 3 Section Clinical Infectious Diseases, University Hospital, LMU Munich, 81377 Munich, Germany; rika.draenert@med.uni-muenchen.de

**Keywords:** meropenem, piperacillin, therapeutic drug monitoring (TDM), critical illness, renal function, pharmacokinetic, experience

## Abstract

Various studies have reported insufficient beta-lactam concentrations in critically ill patients. The extent to which therapeutic drug monitoring (TDM) in clinical practice can reduce insufficient antibiotic concentrations is an ongoing matter of investigation. We retrospectively evaluated routine meropenem and piperacillin measurements in critically ill patients who received antibiotics as short infusions in the first year after initiating a beta-lactam TDM program. Total trough concentrations above 8.0 mg/L for meropenem and above 22.5 mg/L for piperacillin were defined as the breakpoints for target attainment. We included 1832 meropenem samples and 636 piperacillin samples. We found that 39.3% of meropenem and 33.6% of piperacillin samples did not reach the target concentrations. We observed a clear correlation between renal function and antibiotic concentration (meropenem, *r* = 0.53; piperacillin, *r* = 0.63). Patients with renal replacement therapy or creatinine clearance (CrCl) of <70 mL/min had high rates of target attainment with the standard dosing regimens. There was a low number of patients with a CrCl >100 mL/min that achieved the target concentrations with the maximum recommended dosage. Patients with impaired renal function only required TDM if toxic side effects were noted. In contrast, patients with normal renal function required different dosage regimens and TDM-guided therapy to reach the breakpoints of target attainment.

## 1. Introduction

Despite numerous advances in modern medicine, bacterial infections are still associated with high morbidity and mortality in intensive care units (ICUs) [1,2,3]. The quick initiation of effective antibiotic therapy is a decisive factor in treatment success, as this is often the only causal therapy [4,5]. Inadequate drug exposure is one of the primary reasons why the mortality rate in patients with sepsis or septic shock has not decreased significantly over the last 10 years [6]. In particular, hydrophilic drugs, such as beta-lactam antibiotics, show profound pharmacokinetic and pharmacodynamic variability in critical illness due to capillary leakage and altered physiological conditions [7,8,9,10,11,12,13].

Beta-lactam antibiotics exhibit time-dependent antimicrobial activity above the minimal inhibitory concentration (MIC) (*f T_>MIC_*), and recent studies suggest targets of 100% *f T_>MIC_* up to 100% *f T_>8-10xMIC_* to be more appropriate for the critically ill [12,14,15,16]. In this context, prospective studies have shown that approximately every second meropenem and piperacillin measurement fails to achieve defined breakpoints [7,12,17,18]. However, the target level in those studies has been defined on the basis of expert opinions and not on the results of patient outcomes [10,14,19]. 

As both meropenem and piperacillin are mainly eliminated renally, renal function is one of the most influential factors of the concentrations in the bloodstream. Accordingly, young patients with hyperdynamic kidney function are at a higher risk for subtherapeutic concentrations [20,21,22,23]. In contrast, acute kidney failure leads to a significant increase in the half-life of the drugs, with a risk of accumulation and toxic adverse effects [24]. Therefore, several guidelines now recommend beta-lactam therapeutic drug monitoring (TDM) in intensive care patients for the optimization of antimicrobial therapy, particularly for patients who are at risk of unstable renal functions [25,26]. 

Recently, a routine therapeutic drug monitoring program for meropenem and piperacillin was implemented for intensive care unit (ICU) patients at the Department of Anaesthesiology of the University Hospital, LMU Munich, Germany. We retrospectively assessed the frequency of subtherapeutic and toxic concentrations of antibiotics in critical illness in relation to kidney function. In addition, the concentrations were examined over the course of one year to determine to what extent routine beta-lactam TDM and the experience of the attending physicians could improve the target attainment rate within the approved dosage regimens of the summary of product characteristics (SmPC) as short infusions. Furthermore, we aimed to derive dose recommendations for different sectors of renal function for both antibiotics.

## 2. Results

### 2.1. Demographic and Clinical Data

In total, 200 patients treated with meropenem and 89 patients treated with piperacillin were included in the present evaluation (in total, 289 patients). The most frequent reasons for admission to the intensive care unit were (in descending order) lung transplantation, acute respiratory distress syndrome, and liver transplantation. The study population had a median age of 61 years (range 21–97 years), and 68.1% of the patients were male ones, which was in line with the overall patient cohort treated at our ICU. Renal function on the first day of sampling was well preserved in 196 patients without renal replacement therapy, with a median creatinine clearance (CrCl) of 90 mL/min (range 10 to 170 mL/min). The median sepsis-related organ failure assessment (SOFA) score was 12 (range 0 to 24). Detailed demographic and clinical data are given in Table 1.

### 2.2. Assessment of Meropenem Trough Sample Concentration

There were 1834 TDM measurements performed for meropenem. Two samples had to be excluded from the evaluation due to incorrect sampling. For the remaining 1832 samples, 70.2% of the trough levels were obtained for the 1 g (eight-hourly, q8), and 29.8% of the trough levels were obtained for the 2 g (q8) dosage regime. The total median trough level was 10.9 mg/L. The target concentration of ≥8 mg/L was not reached in 39.3% of the samples, and 2.1% of the samples exceeded the toxic breakpoint of 45 mg/L. The median trough concentration for the 1 g (q8) dosing scheme was significantly lower than for the 2 g dosing scheme (*p*-value < 0.01, median 10.6 mg/L versus 11.7 mg/L). The detailed information can be found in Table 2. 

### 2.3. The Impact of Renal Function on Meropenem Concentration

Regardless of renal function, none of the patients received renal dose adjustments, such as 1 g (12-hourly, q12) or 0.5 g (q8). 

The rates of subtherapeutic levels in the different groups based on renal function were as follows: group 1 = 12.4%, group 2 = 11.1%, group 3 = 37.5%, and group 4 = 69.4%. The rates of toxic levels in these groups were: group 1 = 4.5%, group 2 = 7.4%, group 3 = 1.4%, and group 4 = 0.1%. 

Patients in group 1 had high rates of target attainment in both dosing regimens (86.2% after 1 g (q8), 93.3% after 2 g (q8)) and low rates of toxic levels (3.1% after 1 g (q8), 10.1% after 2 g (q8)). In group 2, the trough levels after 1 g meropenem (q8) were occasionally subtherapeutic (13.4%) and sparsely toxic (2.8%) in contrast to 2 g meropenem (q8), which demonstrated many toxic levels (22.1%). In group 3, high rates of target attainment were seen after 2 g meropenem (q8) (83.2%) combined with less toxic levels (3.8%). In group 4, none of the dosing regimens led to sufficient rates of target attainment (17.7% after 1 g (q8), 45.0% after 2 g (q8)). For detailed results, see Table 2. 

The correlation analysis of all meropenem trough levels without renal replacement therapy combined with CrCl showed a strong correlation with *r* = 0.53. The correlation coefficient for 1 g meropenem was *r* = 0.68 and for 2 g meropenem was *r* = 0.58.

**Figure 1:**
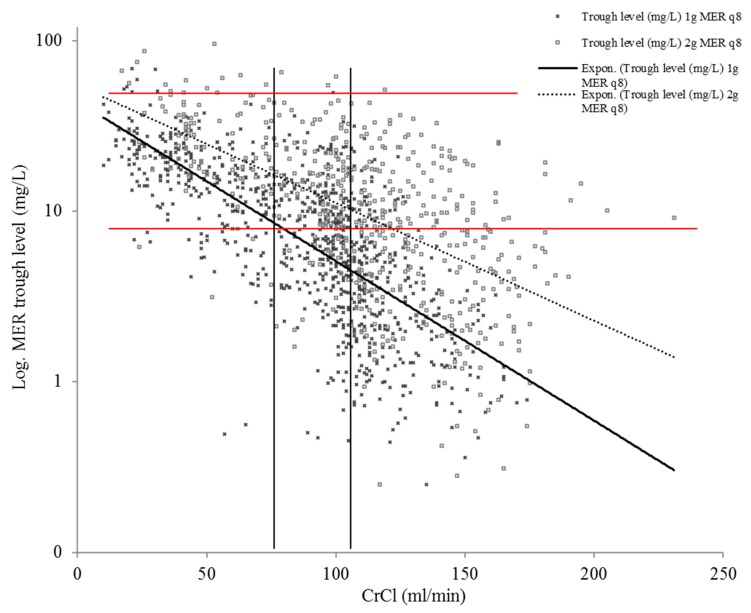
The distribution of meropenem trough level concentrations with the administration of 1 g (q8) and 2 g (q8) in relation to the individual creatinine clearance (CrCl) in patients without renal replacement therapy. The vertical black lines demarcate groups 1–3, and the red horizontal lines denote the breakpoint for target attainment and the toxic breakpoint. Note: log: logarithmic, MER: meropenem, CrCl: creatinine clearance.

Figure 1 shows the distribution of meropenem trough level concentrations in relation to creatinine clearance. 

### 2.4. Assessment of Piperacillin Trough Sample Concentration

There were 644 TDM analyses performed for piperacillin, and eight samples were excluded from the evaluation due to sampling errors. The dosing regimens of piperacillin-tazobactam as a short infusion ranged from two to four times a day. For the remaining 636 samples, 12.6% were quantified after a six-hourly (q6) period, 77.8% after a q8 period, and 9.3% after a q12 period. The total median trough level was 44.3 mg/L. The target concentration of ≥22.5 mg/L was not reached in 33.6%, and 11.0% exceeded the toxic breakpoint of 150 mg/L. The trough concentrations in q6 were significantly lower compared to q12 (*p*-value < 0.01, median 23.5 mg/L versus 65.9 mg/L), and the trough concentrations in q8 were also significantly lower compared to q12 (*p*-value 0.035, median 23.5 mg/L versus 47.1 mg/L). The detailed information can be found in Table 3. 

### 2.5. The Impact of Renal Function on Piperacillin Concentrations

The rates of subtherapeutic levels in the different groups based on renal function were as follows: group 1 = 3.3%, group 2 = 5.6%, group 3 = 43.0%, and group 4 = 74.2%. The rates of toxic levels in these groups were: group 1 = 15.3%, group 2 = 26.2%, group 3 = 2.3%, and group 4 = 0%. 

Patients in group 1 had high rates of target attainment in the q8 dosing scheme (3.6% subtherapeutic, 16.7% toxic) and the q12 dosing scheme (no subtherapeutic, no toxic). In group 2, the trough levels after q12 had lower rates of subtherapeutic levels (4.5%) and a low number of toxic samples (18.2%). In group 3, neither the dosing interval of q8 nor the dosing interval of q6 led to sufficient rates of target attainment (60.6% and 40.0%). Group 4 demonstrated similar results with inadequate rates of target attainment (46.6% after q6, 16.7% after q8). For detailed results, see Table 3. 

The correlation analysis of all piperacillin trough levels without renal replacement therapy combined with CrCl showed a strong correlation with *r* = 0.63. The correlation coefficient for piperacillin (q6) was *r* = 0.56, for piperacillin (q8) was *r* = 0.65, and for piperacillin (q12) was *r* = 0.28.

**Figure 2:**
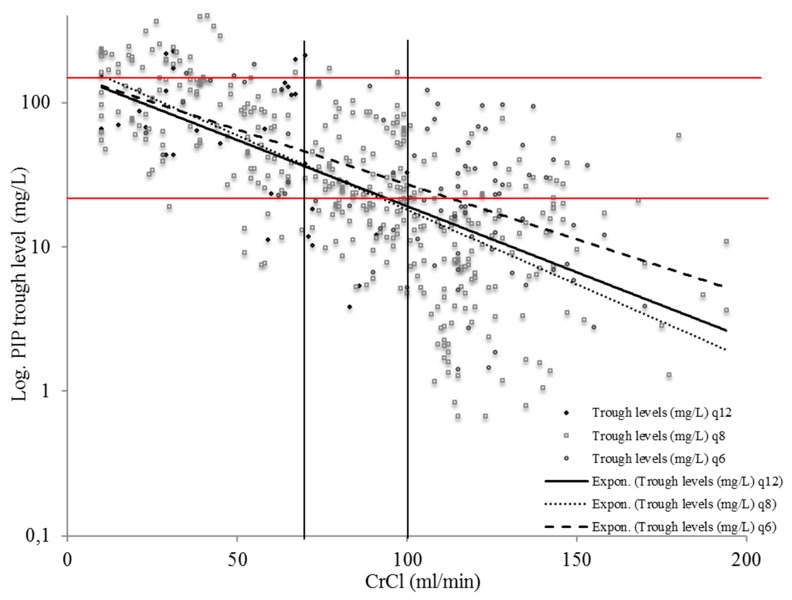
The distribution of piperacillin trough level concentrations with the administration of 4.5 g of piperacillin-tazobactam, twice a day (q12), three times a day (q8), and four times a day (q6) in relation to the individual creatinine clearance (CrCl) in patients without renal replacement therapy. The vertical black lines demarcate groups 1–3, and the red horizontal lines denote the breakpoint for target attainment and the toxic breakpoint. Note: log: logarithmic, PIP: piperacillin, CrCl: creatinine clearance.

Figure 2 shows the distribution of piperacillin trough level concentrations in relation to creatinine clearance. 

### 2.6. Changes in Target Attainment and Dosing Regimens Caused by the TDM-program 

In period 2, the total meropenem median increased from 10.5 to 11.3 mg/L, and the fraction of subtherapeutic trough levels decreased from 40.9% to 38.0%. The portion of 2 g (q8) dosages rose from 19.1% to 41.8% in period 2. Detailed information can be found in Table 2. 

The total piperacillin median increased from 28.3 to 61.5 mg/L, and the fraction of subtherapeutic trough levels decreased from 42.4% to 26.1% in period 2. For detailed information, see Table 3.

### 2.7. Dosing Algorithm for Meropenem and Piperacillin Based on Renal Function

**Figure 3:**
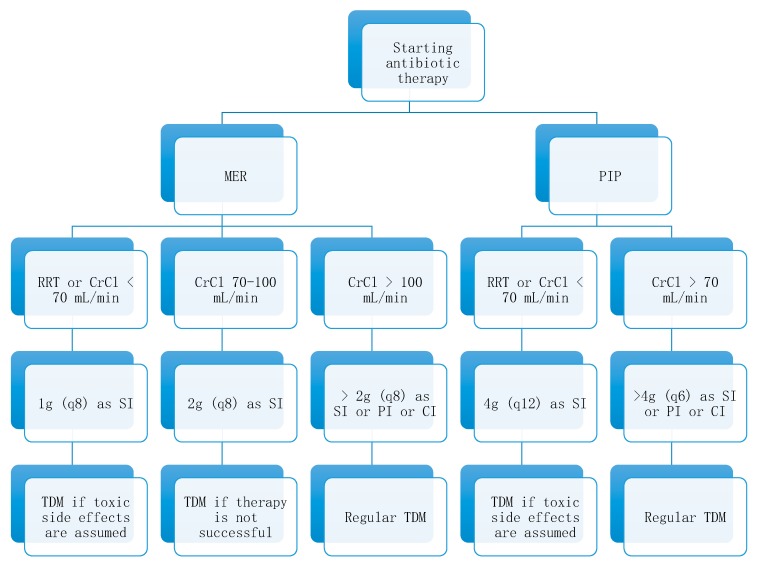
Dosing algorithm for critically ill patients treated with meropenem and piperacillin based on renal function. Note: RRT: renal replacement therapy, CrCl: creatinine-clearance, MER: meropenem, q8: eight-hourly, SI: short infusion = 30 minutes, TDM: therapeutic drug monitoring, PI: prolonged infusion, CI: continuous infusion, PIP: piperacillin, q12: 12-hourly, q6: six-hourly.

Our data showed that renal function was an important influencing factor on beta-lactam antibiotic blood concentration. Dosing algorithms might be helpful in situations when routine therapeutic drug monitoring is not available or as a cost-saving measure. The main condition of a dosing algorithm was that it must be easy to implement, as it should be used in clinical practice. Such an algorithm can be found in Figure 3. 

## 3. Discussion

To the best of our knowledge, this is one of the largest retrospective analyses of routine beta-lactam TDM in intensive care patients [27,28,29]. Many societies and authors recommend a daily TDM of beta-lactams in critically ill patients [16,25,28,30]. In this study, we examined for whom daily TDM is necessary, for whom a short infusion-dosing regimen is suitable, and which patients need other dosing strategies. 

On average, 40% of meropenem samples did not achieve a sufficient trough concentration of 8 mg/L. We thought that the chosen high breakpoints for target attainment were necessary due to the high number of immunocompromised patients who underwent lung or liver transplantation and had special pathogens with higher MICs than other patients [31,32]. The number of subtherapeutic levels was comparable to those reported in the literature for meropenem [17,33,34], piperacillin [7,35,36], and other beta-lactam antibiotics like cefepime [37] or ceftriaxone [38]. Furthermore, the existing correlation between renal function and beta-lactam blood concentration has also been described in different studies [21,22].

Typically, beta-lactam dosage should be adapted based on renal function. However, there are no standardized breakpoints for dose adaption. The German SmPC, for example, recommends a dose reduction for meropenem if CrCl <50 mL/min [39] and for piperacillin if CrCl <40 mL/min [40]. Nevertheless, these recommendations build on patients with chronic kidney disease and are not suitable for intensive care patients with acute kidney injury. Hence, other breakpoints for dose adaption should be used. 

The criteria for an appropriate breakpoint of dose adaption in intensive care unit patients are high rates of target attainment and low rates of toxic levels. Therefore, we decided to use the breakpoints of CrCl <70 mL/min or renal replacement therapy (RRT), CrCl 70–100 mL/min, and CrCl >100 mL/min. Other authors in similar settings have also used different breakpoints like CrCl <50 mL/min and >100 mL/min [41] or CrCl <60 mL/min and >120 mL/min [42]. 

We observed that up to a CrCl of <70 mL/min or renal replacement therapy, a dosage of 1 g meropenem eight-hourly as a short infusion was sufficient in the majority of cases, whereby a dosage of 2 g meropenem eight-hourly led to high rates of toxic levels (22.1% for group 2 and 10.1% for group 1) with the risk of nephrotoxicity and neurotoxicity [43,44]. For patients with a CrCl of 70–100 mL/min, a dosage of 2 g meropenem eight-hourly was necessary to achieve the therapeutic range. Daily TDM is, regarding economic policy, not necessary if renal function is consistent. In patients with a CrCl >100 mL/min, high rates of target non-attainment were observed for both dosing regimens, so they must be considered insufficient. 

Augmented renal clearance is a well-known risk factor for target failure in antibiotic dosing [35,45], and a number of published studies have shown that prolonged infusion is a valuable treatment option with respect to target attainment, pathogen eradication, and the reduction of mortality [34,46,47]. Nevertheless, many treating physicians in Germany, where the SmPC does not support prolonged infusion, feel uncomfortable with this type of treatment. In addition, Carlier et al. described that prolonged or continuous infusion with the highest supported dosage of six grams of meropenem per day was not enough to achieve therapeutic levels [48]. Hence, prolonged or continuous infusion should be used for those patients, and in cases of target non-attainment, with higher than the recommended dosage [20,49].

Approximately one-third of all piperacillin trough samples did not reach the target. Similar to meropenem, there was also a higher risk for subtherapeutic trough levels with increasing CrCl (*r*-score >0.6). Patients with a CrCl <70 mL/min or renal replacement therapy had high rates of target attainment with 12-hourly dosing. In contrast, 28.2% of the patients with eight-hourly dosing and a CrCl <70 mL/min had toxic trough levels. Toxic side effects are often underestimated [44], and frequent TDM might be necessary for this patient group to lower the toxic side effects. 

Neither the q8 nor the q6 dosing regimen led to a tolerable rate of target attainment in patients with CrCl >70 mL/min. One reason might be that the only option to adjust the therapy was to shorten the dosing interval. The target levels were most frequently missed in the q6 dosage regimen, presumably due to augmented renal clearance in those patients. For them, other dosing regimens like continuous infusion are necessary to achieve the target [50]. Richter et al. reported higher rates of target attainment and reduced mortality in patients treated with continuous piperacillin infusion in contrast to a short infusion regimen [51].

Indeed, target non-attainment is not only a problem in the therapy with beta-lactam antibiotics. It can also be seen in the therapy with other antibiotic classes like linezolid [52] or ciprofloxacin [53], where standard dosing regimens lead to insufficient concentrations in critically ill patients. A routine therapeutic drug-monitoring program is also useful for these drugs. We still developed a method for the quantification of these drugs [54], and we would expand the current TDM-program soon to further optimize antibiotic therapy at our ICU. 

Focusing on the two periods, higher rates of target attainment and higher medians were observed for both antibiotics in the second half of the year. There were no special instructions for the attending physicians or new standard operating procedures (SOPs) after initiating the TDM-program, explaining the relatively small changes. We assumed that these changes were attributable to the growing experience of the physicians with the TDM-program. Nevertheless, our results suggested that special instructions and SOPs were needed after initiating a TDM-program, especially at large ICUs with many physicians. 

There were limitations to the present study. Due to the retrospective setting, there were no regulations for the antibiotic TDM requests and no requirements for dose adjustment. The data set represented the true situation as it was observed in everyday clinical life in intensive care units, where several doctors often participate in the therapeutic process. Despite meticulous examination, we could not rule out that a minor proportion of falsely designated trough level samples were included in the present evaluation. Our recommendations for different dosage regimens depending on renal function were based on retrospective data. Unlimited application to other patients was not possible. Therefore, prospective data must be generated. 

## 4. Materials and Methods 

### 4.1. Study Setting

This was a monocentric, retrospective study, investigating the appropriateness of short infusion dosage regimes of meropenem and piperacillin in ICU patients with a routine antibiotic TDM program. Clinical and laboratory parameters, including beta-lactam antibiotic serum concentrations, were documented between January and December 2018. The study was conducted in accordance with the Declaration of Helsinki, and the local Ethics Committee (registration number 18-578) approved the protocol. The study was registered at clinicaltrials.gov (NCT03985605).

### 4.2. Study Population

Patients aged ≥18 years, admitted to the ICU, treated with either meropenem or piperacillin-tazobactam, and subjected to TDM in routine clinical practice were eligible for study inclusion. Patients were only enrolled when at least three individual serum trough samples were taken in the pharmacokinetic steady state. Patients were excluded if less than three trough samples were subjected to antibiotic TDM.

### 4.3. Drug Administration, TDM, and Data Collection

The dosage of meropenem and piperacillin administered was at the discretion of the treating physician with varying doses of 1 or 2 g of meropenem given 8-hourly (q8) and 4.5 g piperacillin-tazobactam given 6-, 8-, or 12-hourly (q6, q8, or q12), with each dose administered intravenously over 30 min (denoted as short infusion). No other dosing regimens like prolonged infusion were used due to the German SmPC and the resulting standard operating procedure at our intensive care units. Demographic data and clinical variables, such as vital parameters and weight and organ-support extracorporeal therapies, were collected from the medical record. In addition, different laboratory variables were collected from the laboratory information system. Based on these values, the sepsis-related organ failure assessment (SOFA) score was calculated on the first day of blood sampling. The creatinine clearance (CrCl) was calculated with Chronic Kidney Disease Epidemiology Collaboration (CKD-EPI) [55]. Patients were categorized in different groups depending on their renal function as follows: group 1: renal replacement therapy, group 2: CrCl <70 mL/min, group 3: CrCl 70–100 mL/min, and group 4: CrCl >100 mL/min. 

Trough levels and dosing schemes of the first half of the year after initiation of the TDM program (denoted as period 1) were compared with the second half of the year (denoted as period 2). 

### 4.4. Sample Collection and Laboratory Testing

Meropenem and piperacillin trough blood samples were immediately transferred to the laboratory and centrifuged at 2000 *g* for 10 min. The serum supernatant was subjected to a TDM analysis with a previously published isotope dilution HPLC tandem mass spectrometry (HPLC-MS/MS) method [54,56]. The general clinical chemical parameters were determined with standard clinical chemical methods.

### 4.5. Pharmacokinetic/pharmacodynamic Targets

Non-species-related (European Committee on Antimicrobial Susceptibility Testing (EUCAST) breakpoints were used as the lower limit of the therapeutic range. For the attending physicians, the target concentration for meropenem was 100% *f T_>4xMIC_* (>8 mg/L) due to the clinically sensible breakpoint against the pathogenic *Pseudomonas* spp. at 2 mg/L [57]. Trough levels above 45 mg/L were defined as toxic levels [43]. The target steady-state concentration for piperacillin was 100% *f T_>MIC_* (>22.5 mg/L total drug concentration, assuming an average protein binding of 30%) due to the clinically sensible breakpoint against the pathogenic *Pseudomonas* spp. at 16 mg/L [57]. In accordance with the published literature, toxic piperacillin levels were defined as trough concentrations above 150 mg/L [58]. *Pseudomonas* spp. was used as the reference pathogen as it is associated with high mortality in immunocompromised patients [59].

### 4.6. Statistical Analysis

Measurement results for inadequately documented drug administration or erroneously collected blood samples were excluded from the evaluation. Exclusion criteria were blood sampling during antibiotic administration and measurement despite the cessation of antibiotic therapy. Statistical analysis was performed with IBM SPSS statistics (IBM Corp. Released 2018. IBM SPSS Statistics for Windows, Version 26.0. Armonk, NY, USA: IBM Corp.). The data set was evaluated with respect to the distribution of antibiotics trough sample concentrations for different dosage regimens, the impact of creatinine clearance on drug concentrations, and the changes during the year. Estimates of significance between different CrCl subgroups, dosage regimens, and time periods were obtained with the Mann–Whitney U-test and defined as significant by using a p-value of <0.01. The coefficient of correlation (r) was calculated using a Pearson correlation analysis.

## 5. Conclusions

Daily therapeutic drug monitoring of meropenem and piperacillin in critically ill patients is necessary when their renal function is normal or augmented. Furthermore, a short infusion regimen is not suitable for this group. At our ICU, patients with impaired renal function or renal replacement therapy had high rates of target attainment with standard dosing; however, therapeutic drug monitoring is necessary if toxic side effects are assumed. Experience in the TDM-program leads to higher rates of target attainment in the second half of the year. 

## Figures and Tables

**Table 1:** Demographic and clinical data.

Variable	*n* (%) or Median (Interquartile-Range)
Age (years)	61 (48–73)
Sex (male/female)	190/99
Weight (kg)	74 (64–87)
BMI (kg/m^2^)	24.7 (21.3–28.5)
SOFA Score on the first day of sampling	12 (8–14)
Serum creatinine on the first day of sampling	1.0 (0.8–1.7)
CrCl on the first day of sampling, mL/min	90 (73–113)
CrCl <45 mL/min on the first day of sampling	30 (15.3)
CrCl 45–90 mL/min on the first day of sampling	58 (29.6)
CrCl >90 mL/min on the first day of sampling	108 (55.1)
RRT	83 (28.7)
LETX	27 (9.3)
LTX	96 (33.2)
ARDS	31 (10.7)

Note: BMI: body mass index; SOFA: sepsis-related organ failure assessment; CrCl: creatinine clearance, RRT: renal replacement therapy; LETX: liver exclusive intestinal transplant; LTX: lung transplant; ARDS: acute respiratory distress syndrome.

**Table 2:** Meropenem dosing, meropenem concentration based on renal function, and statistical differences in different groups.

TDM			Samples (%)		Subtherapeutic (%)		Toxic (%)		Median (mg/L) (IQR)		Statistic
All			100		39.3		2.1		10.9 (4.9–19.5)		
1 g (q8)			70.2		40.8		1.3		10.6 (4.6–17.9)		Lower than 2 g (q8) (*p* < 0.01, *r* = 0.18)
2 g (q8)			29.8		35.0		3.8		11.7 (5.8–27.5)		Higher than 1 g (q8) (*p* < 0.01, *r* = 0.18)
Group 1	all		24.3		12.4		4.5		17.4 (11.4–21.9)		
	1 g (q8)	2 g (q8)	19.4	4.9	13.8	6.7	3.1	10.1	16.4 (11.4–21.9)	30.8 (19.2–38.6)	
Group 2	All		17.7		11.1		7.4		19.6 (14.1–27.5)		
	1 g (q8)	2 g (q8)	13.5	4.2	13.4	3.9	2.8	22.1	17.9 (12.7–24.5)	28.7 (18.2–41.9)	
Group 3	All		19.0		37.5		1.4		9.9 (5.6–14.8)		
	1 g (q8)	2 g (q8)	13.2	5.8	46.7	16.8	0.4	3.7	8.6 (4.9–12.7)	15.0 (9.3–23.6)	
Group 4	All		39.0		69.4		0.1		4.5 (2.1–9.2)		
	1 g (q8)	2 g (q8)	20.6	18.4	82.3	55.0	0	0.3	3.3 (1.7–6.1)	7.2 (3.5–13.3)	
Period 1	All		45.3		40.9		2.8		10.5 (4.2–19.1)		No sig. difference to period 2 (*p* = 0.08, *r* = 0.04)
	1 g (q8)	2 g (q8)	36.7	8.6	41.8	37.3	1.6	7.6	10.3 (4.1–18.7)	10.9 (4.6–23.9)	
Period 2	All		54.7		38.0		3.1		11.3 (5.3–19.6)		No sig. difference to period 1 (*p* = 0.08, *r* = 0.04)
	1 g (q8)	2 g (q8)	31.8	22.9	41.8	32.7	1.9	4.8	9.9 (4.7–16.7)	13.3 (6.3–26.7)	

Group 1: renal replacement therapy; Group 2: CrCl <70 mL/min; Group 3: CrCl 70–100 mL/min; Group 4: CrCL >100 mL/min; Period 1: first half of the year; Period 2: second half of the year; q8: eight-hourly; IQR: interquartile range, TDM: therapeutic drug monitoring.

**Table 3:** Piperacillin dosing, piperacillin concentration based on renal function, and statistical differences in different groups.

TDM				Samples (%)			Subtherapeutic (%)			Toxic (%)			Median (mg/L) (IQR)			Statistic
All				100			33.6			11.0			44.3 (15.7–94.4)			
q6				12.6			45.0			10.2			23.5 (12.1–51.2)			Lower than q12 (*p* < 0.01, *r* = 0.34)
q8				77.8			33.8			12.3			47.1 (15.7–96.5)			Lower than q12 (*p* = 0.04, *r* = 0.1),
q12				9.3			18.6			3.7			65.9 (40.7–120.5)			Higher than q8 (*p* = 0.04, *r* = 0.1) and q6 (*p* < 0.01, *r* = 0.34)
Group 1	all			24.8			3.3			15.3			90.6 (66.6–127.5)			
	q6	q8	q 12	0	22.9	1.9	/	3.6	0	/	16.7	0	/	91.4 (67.2–128.7)	77.3 (50.8–121.5)	
Group 2	all			25.2			5.6			26.2			94.1 (52.0–154.0)			
	q6	q8	q 12	2.2	19.5	3.5	0	5.8	4.5	21.4	28.2	18.2	63.0 (47.8–142.2)	94.1 (50.8–162,2)	79.0 (64.8–127.7)	
Group 3	all			20.1			43.0			2.3			25.4 (15.3–47.3)			
	q6	q8	q 12	1.6	17.1	1.4	60.0	39.4	66.7	0	1.8	11.1	20.1 (13.2–31.5)	25.9 (17.8–51.3)	12.2 (10.3–32.7)	
Group 4	all			29.9			74.2			0			12.1 (5.1–23.1)			
	q6	q8	q 12	9.1	20.8	0	53.4	83.3	/	0	0	/	6.6 (9.1–37.5)	8.2 (3.8–17.2)	/	
Period 1	all			46.4			42.4			6.4			28.3 (12.7–80.6)			Lower than period 2 (*p* < 0.01, *r* = 0.2)
	q6	q8	q 12	4.7	41.7	0	50.0	41.5	/	0	7.2	/	22.0 (10.1–30.8)	30.7 (14.0–81.5)	/	
Period 2	all			53.6			26.1			15.0			61.5 (20.9–114.0)			Higher than period 1 (*p* < 0.01, *r* = 0.2)
	q6	q8	q 12	7.9	36.5	9.2	44.0	24.5	16.9	6.0	18.1	10.2	29.2 (13.5–64.7)	67.4 (23.3–124.5)	64.6 (40.7–117.5)	

Note: Group 1: renal replacement therapy; Group 2: CrCl <70 mL/min; Group 3: CrCl 70–100 mL/min; Group 4: CrCL >100 mL/min; Period 1: first half of the year; Period 2: second half of the year; q6: six-hourly; q8: eight-hourly; q12: twelve-hourly; IQR: interquartile range, TDM: therapeutic drug monitoring.
